# Living with myotonic dystrophy; what can be learned from couples? a qualitative study

**DOI:** 10.1186/1471-2377-11-86

**Published:** 2011-07-13

**Authors:** Edith HC Cup, Astrid Kinébanian, Ton Satink, Allan J Pieterse, Henk T Hendricks, Rob AB Oostendorp, Gert Jan van der Wilt, Baziel GM van Engelen

**Affiliations:** 1 Nijmegen Centre for Evidence Based Practice, Department of Rehabilitation/Occupational Therapy 898, Radboud University Nijmegen Medical Centre, P.O. Box 9101, 6500 HB, Nijmegen, The Netherlands; 2 Leusdenhof 100, 1108 CZ Amsterdam, the Netherlands; 3 Department of Occupational Therapy and European Masters of Science in Occupational Therapy, HAN University of Applied Sciences, c/o Kapittelweg 33, 6525 EN Nijmegen, The Netherlands; 4 Nijmegen Centre for Evidence Based Practice, Department of Rehabilitation/Physical Therapy 898, Radboud University Nijmegen Medical Centre, P.O. Box 9101, 6500 HB Nijmegen, The Netherlands; 5 Groot Klimmendaal Arnhem, P.O. Box 9044, 6800 GG Arnhem, the Netherlands; 6 Nijmegen Centre for Evidence Based Practice, Scientific Institute for Quality of Healthcare, 114, Radboud University Nijmegen Medical Centre, P.O. Box 9101, 6500 HB Nijmegen, The Netherlands; 7 Nijmegen Centre for Evidence Based Practice, Department of Epidemiology, Biostatistics and HTA 133, Radboud University Nijmegen Medical Centre, P.O. Box 9101, 6500 HB Nijmegen, The Netherlands; 8 Department of Neurology 935, Donders Institute for Brain, Cognition and Behaviour, Radboud University Nijmegen Medical Centre, P.O. Box 9101, 6500 HB Nijmegen, The Netherlands

## Abstract

**Background:**

Myotonic dystrophy type 1 (MD1) is one of the most prevalent neuromuscular diseases, yet very little is known about how MD1 affects the lives of couples and how they themselves manage individually and together. To better match health care to their problems, concerns and needs, it is important to understand their perspective of living with this hereditary, systemic disease.

**Methods:**

A qualitative study was carried out with a purposive sample of five middle-aged couples, including three men and two women with MD1 and their partners. Fifteen in-depth interviews with persons with MD1, with their partners and with both of them as a couple took place in the homes of the couples in two cities and three villages in the Netherlands in 2009.

**Results:**

People with MD1 associate this progressive, neuromuscular condition with decreasing abilities, describing physical, cognitive and psychosocial barriers to everyday activities and social participation. Partners highlighted the increasing care giving burden, giving directions and using reminders to compensate for the lack of initiative and avoidant behaviour due to MD1. Couples portrayed the dilemmas and frustrations of renegotiating roles and responsibilities; stressing the importance of achieving a balance between individual and shared activities. All participants experienced a lack of understanding from relatives, friends, and society, including health care, leading to withdrawal and isolation. Health care was perceived as fragmentary, with specialists focusing on specific aspects of the disease rather than seeking to understand the implications of the systemic disorder on daily life.

**Conclusions:**

Learning from these couples has resulted in recommendations that challenge the tendency to treat MD1 as a condition with primarily physical impairments. It is vital to listen to couples, to elicit the impact of MD1, as a multisystem disorder that influences every aspect of their life together. Couple management, supporting the self-management skills of both partners is proposed as a way of reducing the mismatch between health services and health needs.

## Background

"Every time I ask her to come shopping with me, she says you better go without me".

This quote is typical for a partner of someone with myotonic dystrophy type 1 (MD1). It may raise questions like why this person with MD1 chooses to stay at home? What does it mean for her and her partner? Is it considered a problem or not and if so by whom? These questions address the impact of MD1 on daily life of couples and are related to their health and well-being. How often are such issues addressed by health care professionals?

MD1, also known as Steinert disease, is the most frequent of the adult neuromuscular diseases with a prevalence estimated at 1/20,000 inhabitants [1]. MD1 is a hereditary chronic systemic disease with wide variability in clinical expression, both within and between families. MD1 is characterized by the involvement of many organs, including impairments in the muscular, respiratory, cardiac, central nervous, endocrine and ocular functions and structures [2-4]. Typical symptoms of the disease include myotonia and progressive loss of muscle strength, usually distal to proximal, and weakness of facial and anterior neck muscles. Other symptoms include cataracts, frontal baldness, dysarthria, fatigue and daytime somnolence. Also cognitive decline including lack of initiative and specific personality traits such as an avoidant personality are related to MD1 [5-8]. Compared to other neuromuscular diseases, persons with MD1 have among the gravest functional disabilities and the greatest dependence on others for activities of daily living [9]. They also have the lowest social participation, most psychosocial problems and poorest psychosocial well-being [10]. This not only has huge impact on persons having the disease [8,10,11], as well as on their partners [12,13] and surely on their lives together as a couple. As far as we know, this is the first study to explore the impact of MD1 on the daily lives of couples.

Despite the huge impact of MD1 on daily life, individuals with MD1 tend to play down or avoid expressing their problems when attending physicians or other health care professionals and adherence to rehabilitation services is generally poor [14]. Reasons for this are not well understood. Difficulties managing their own health has been attributed to personality features such as diminished persistence and increased avoidance [5,15,16]. It may also be that persons with MD1 perceive their limitations as normal, since the slowly progressive nature of the deterioration gives them time to adapt and live more by what they can do than by what they cannot do [10] or they might have received the message that the disease is progressive and that there is no cure [8]. Finally, they may believe that professionals cannot help and have little knowledge and understanding of the disease [8]. Indeed, although MD1 is one of the most prevalent neuromuscular diseases, the disease is relatively unknown among medical and allied health professionals and scientific evidence of the effectiveness of allied health care is limited as shown in the various systematic reviews [17-19]. Very little is known about how couples cope in daily life with this complex, progressive disease. It is important to understand their perspective of living with this hereditary, systemic disease in order to better match health care to their problems, concerns and needs. Persons with MD1 and their partners both live with the disease and become experts through their experience. They are more knowledgeable than professionals regarding the consequences of the disease for their lives and how treatment effects their daily lives [20,21].

The purpose of this study is to increase our understanding of what MD1 means for persons having the disease, for their partners and for both as a couple, and how they manage in daily life individually and together. The research questions are:

- How do persons with MD1 and their partners reflect on living with this hereditary chronic progressive disease?

- How do persons with MD1 and their partners experience their lives together as a couple?

## Methods

### Design

As we aim to study personal experiences, make sense of these and interpret these in terms of the meanings the persons themselves bring to them, a qualitative method is most appropriate [22]. Couples were visited in their homes. In 2009, 15 in-depth interviews were carried out with persons with MD1 and their partners separately and simultaneously at two different moments in time to obtain insights in the meaning of MD1 on the lives of couples.

This study followed a hermeneutic approach according to the method of '*verstehen*', which is based on three principles [22]:

1) Human experience has a historical nature and is context bound. This was addressed by asking the participants about present and past experiences and the context of these and by conducting the interviews in the homes of the couples, their naturalistic setting.

2) Understanding requires imaginative participation in the lives of those being studied to not impose foreign meanings on their thoughts and actions. This was addressed by listening to the couples and observing how they live and manage in their home situation.

3) The researchers' professional perspectives and experiences are used when interpreting the findings to gain better insight and understanding of the meaning of living with MD1.

### Data collection and procedure

The researcher (EC) approached all couples by telephone. All agreed to receive written information about the study. When contacted a week later, all couples agreed to participate in the study. The first couple served as a pilot couple to test the interview guide (Table 1). As lack of understanding was a major issue raised spontaneously by this couple, this topic was added to the interview guide.

**Table 1:** Interview guide


**Main question**
"How has myotonic dystrophy influenced your daily life and how do you manage?"

**Topics**
Current daily activities, roles and relations and their meaning
Activities, roles and relations and their meaning in the past
Changes in activities roles and relations
Management strategies to deal with changes

**Topic added following the pilot interview**
Understanding from others

Data collection was iterative and consisted of four phases for each couple:

1) Individual interview with the person having the MD1;

2) Individual interview with the partner;

3) Verbatim transcription of interviews and selection of meaningful expressions or topics by the researcher (EC);

4) Interview with couple about three weeks later. The goal was to elaborate on previous topics and to get more in depth information on how MD1 affects the lives of couples and how they manage together.

All persons were interviewed in the room and chair where they felt most comfortable and were they could speak freely in a confidential situation. The individual interviews with the person with MD1 and with the partner took place without the other being present in the same room. Small talk and an explanation of the purpose and format of the interview enabled the interviewer and interviewees to become acquainted with each other. The interview guide comprised one main question and follow-up topics. Each interview proceeded in an informal conversational style, lasting between 60 and 90 minutes. During the visit the interviewer gained an impression how persons with MD1 and their partners lived and managed individually and together, by observing how they moved around in the house and made a drink or something to eat. These were random observations depending on the situation and the kind of activities the persons had reason to do. Following the interviews, the researcher wrote memos on the impressions and reflections of the interview and field notes regarding the observation of how the persons managed at home.

### Ethical considerations

Preceding the interviews, informed consent forms were signed. At all visits the researcher emphasized that participation was entirely voluntary and that the participants could withdraw at any time without consequences for their treatment. Permission to audiotape the interview was confirmed at every visit as was reassurance of confidentiality. Following the interviews, the researcher gave participants an opportunity to reflect on the experiences and checked the need for formal after care from a social worker. None of the participants indicated they needed this. All data and quotes used were anonymized. Ethical approval was obtained from the local medical ethics committee (CMO number 2008/261 NL26868.091.09).

### Participants

Ten persons, five with MD1 (adult or classical type) and their partners, were included. Purposive sampling [23] was used, identifying couples with MD1 from the Dutch neuromuscular database, CRAMP (Computer Registry of All Myopathies and Polyneuropathies) which contains about 300 persons with MD1. All participants with MD1 had been admitted to the medical centre in the past for the purpose of multidisciplinary (medical and allied health) assessment and advice. Criteria for inclusion were 1) the person with MD1 was restricted in activities of daily living and social participation; and 2) the couples had been living together for at least ten years. Difficulties in communication such as dysarthria, which had been an exclusion criterion in other qualitative studies with persons having MD1 only, was not an exclusion criterion in the current study.

Three men and two women with MD1 and their partners, all Caucasian, were willing to participate. They lived in different villages in the south of the Netherlands. The mean age of the persons with MD1 was 58 years (range 53 - 65). In all persons with MD1 the onset of the symptoms was in adulthood. They all experienced problems with mobility, one was confined to a wheelchair. There was variation in socio-economic background and work status. (Table 2).

**Table 2:** Characteristics of participating couples

Couple	Age person with MD1	Gender person with MD1	Work status person with MD1	Work status partner
1	65 yrs	Male	Early retirement	Housewife
2	53 yrs	Male	Sheltered work, part time	Sheltered work, part time
3	58 yrs	Male	Disability pension/retired	Working on occasions
4	56 yrs	Female	Housewife	Early retirement
5	56 yrs	Female	Housewife	Working full time

MD1 = myotonic dystrophy type 1; yrs = years

### Data analysis

Analysis started following initial interviews with each couple. In depth analysis took place after completing all 15 interviews. Analysis involved a constant comparison method [24] including the following steps:

1) Familiarization with the interviews and understanding of the experiences and meaning of living with MD1 by listening and multiple readings of the transcripts, the production of summaries and reading the reflexive memos and field notes;

2) Assignment of codes to meaningful text units (statements) related to the research questions. In-vivo codes were assigned to stay close to the original voices of the interviewees.

3) Categorization of meaning units from the combination of all codes, reorganization and interpretation, resulting in the formulation of themes and subthemes; and

4) Interpretation of relations between themes and subthemes with use of other sources including literature, field notes, reflections and own experiences leading to new insights and better understanding of the meaning of MD1 for couples.

The researcher (EC) performed all steps and two co-authors (TS, AK) served as peer-reviewers during the process of coding and the formulation of themes and subthemes. The codes, themes and subthemes were discussed with the whole research group at three meetings. MaxQDA 2007 computer software was used to assist in organizing the data http://www.MAXQDA.com. The analysis was carried out in the Dutch language to minimize bias and preserve meaning. Only the quotations used in this article were translated into English.

### Reflection on the role of researcher and research group

The research group included researchers with different medical, philosophical, allied health care and methodology background. Three members were experienced researchers and lecturers in the field of qualitative research (AK, TS, GJW). The researcher (EC) also worked as an occupational therapist in a multidisciplinary team of clinicians and researchers. Previously, she had seen three of the participants with MD1 for a single assessment and advice in the neuromuscular centre. In the restricted time allocated in a clinical setting, she had felt it was impossible to understand what it is like for couples living with MD1. The fact that the researcher had met three people with MD1 once before did not seem to influence the participants in sharing their personal experiences.

### Trustworthiness

To further enhance the trustworthiness or rigor of the study [25], several triangulation methods were used [26], including the use of different perspectives (persons with MD1, their partners and as a couple) generating richer understanding than single perspectives [27]; and investigator triangulation within the research group. Credibility [25] of the study was further ensured by member checking and the use of participants' quotes and in-vivo (sub) themes. All couples had received summaries of the interviews including meaningful quotes and had confirmed content.

## Results

Four themes emerged from the interviews representing the impact for persons with MD1, for their partners, and for both of them as a (married) couple, and the last theme represented an overall impact (Table 3). The subthemes reflect the impact of living with MD1 and the main self-management strategy used. To capture the essence of a (sub) theme, in vivo codes were used. Most codes were related to the person with MD1 and least related to partners. Persons with MD1 gave less information on their partners whereas partners gave much information related to persons with MD1.

**Table 3:** The four themes resulting from the analysis

Impact for persons with MD1
1) Decreasing abilities
• *Many barriers; "no power, no pep, no guts"*
• *Avoiding barriers; "I don't do that much"*
Impact for partners
**2) Increasing burden**
• *"If I did not do it, nothing happened"*
• *"I am in charge; I give directions*"

Impact for both as a (married) couple
**3) Finding a mode together**
• *Doing or leaving; renegotiation of tasks*
• *Individual and shared activities; give each other freedom*

Impact for persons with MD1, for partners and for couples **4) Lack of understanding**
• *Even the family does not understand*
• *We manage ourselves*
• *Health care: many islands focused on part of the disease*

### Theme 1: Decreasing abilities

All persons with MD1 experienced progressively more limitations in an increasing number of daily activities and roles which were important to them. These activities were enjoyable, made them independent, gave a sense of belonging or made them feel valued by others. For some persons the decrease in abilities felt like "I cannot do anything anymore".

"I have always helped my brothers a lot. Those were good times. I could do it all. But that's history. I cannot do anything anymore. Now they no longer need me. They have sort of forgotten me." [couple 1: male with MD1]

#### Many barriers; no power, no pep, no guts

Persons with MD1 experienced obstacles to everyday activities and social participation. Some referred to barriers; others said it was too much trouble, too much hassle, too difficult, or too complicated. When someone said that he or she lacked pep or guts, it could mean that he or she lacked energy or vitality (physical barrier), experienced difficulty initiating or planning tasks (cognitive barrier), or lacked courage (psychosocial barrier) or a combination thereof.

"For a lot of things I just don't have the guts. Then it takes ages before I get dressed, simply because I don't feel like it." [couple 4: female with MD1]

Persons with MD1 reported physical limitations such as reduced strength in hands and legs, decreased balance, reduced fitness and fatigue. These limitations led to safety risks, fear of falling, inability or a disproportionate amount of time or effort was needed to perform tasks.

"I used to set the table. But I cannot do this anymore. Because, when I take the plates and I fall, then we have no plates anymore..." [couple 2: male with MD1]

Observation of this person manoeuvring around in his home showed the difficulties he encountered.

"My condition is nearly nothing. I cannot walk anymore, at least, not what it should be. When I walk, I'm dead tired. I walk as little as possible." [couple 1: male with MD1]

Several participants mentioned problems with their bowels or continence which restricted participation in daily activities.

"I used to sit on the toilet the whole morning. Now I notice with my bladder sometimes..... I cannot do much, otherwise I may lose control." [couple 4: female with MD1]

Few participants described lack of initiative or mentioned that thinking what to do or how to do it was difficult for them. Instead, many regarded themselves as 'easy' and some wondered whether they might be too easy going.

"Deciding what we are going to eat is terribly difficult for me." [couple 3: male with MD1]

"Persons with my muscle disease don't have much initiative. If my wife suggests shall we go on holiday? Then I say okay, you say where, because I don't care. She does not like that. She says you can think of what you like. Then I say I enjoy everything, I am very easy." [couple 3: male with MD1]

In social situations, persons with MD1 found it hard to take initiative and usually waited for invitations from others. They described barriers associated with feelings of shame or insecurity. The reduced intelligibility or clumsiness made them feel anxious and led to fear of being looked at or being made a fool of. One participant used to sit in her chair near the window during the day, hoping that her daughter or grandchildren would visit or waiting for her husband to come home.

"On a birthday, I'll quietly sit in a corner. I dare not say anything. There are also strange people that might not understand me or make me feel foolish." [couple 5: female with MD1]

#### Avoiding barriers; I don't do that much

Persons with MD1 mentioned that they increasingly give up, postpone or avoid activities and roles, like stopping work or sports and going out less. Even the daily shopping is often left to the partner. The result is that their world becomes smaller and social contacts decline. Although this is perceived as a difficult and painful process, the participants felt that there is no use in complaining and tried to stay positive.

"I get very annoyed when I'm in a shop and I cannot open a bag to put tomatoes in it or something. Then I have to ask to keep the bag open. It may be false modesty or shame, but I find that terrible. That's why I have quitted doing the shopping." [couple 4: female with MD1]

"Because you are at home most of the time, your world becomes smaller, which is very annoying for me..... But I am not going to sit and be sad. I still do things, but not that much." [couple 3: male with MD1]

Participants also adjusted their expectations and demands, making life more manageable. Sedentary or spectator activities were mentioned like enjoying the presence of grandchildren or sitting in the garden or at the camping site. They described adapting activities or doing less complicated activities in order to participate with less effort.

"I'm short of breath. When I sing, I occasionally cheat, I call it playback. We have performances, which is nice and it is also good for social contacts. " [couple 1: male with MD1]

Participants found alternatives like playing bridge instead of tennis or joining a book club instead of the quilt club. Being a member of a club or a group of friends with regular appointments appeared very helpful to stay in touch with others and have fun. Participants mentioned that they perceived no barriers when doing sedentary activities such as reading or watching television and experienced much pleasure doing this.

"Television is important to me. Especially since I've always been a sports fan, I like to watch sports. Soon Wimbledon and Tour de France are on television. Then I watch television in the afternoon. For me it's an important thing to relax." [couple 2: male with MD1]

### Theme 2: Increasing burden

Partners expressed how they gradually had taken over more activities or even the entire organization of their lives as a couple. Because it happens gradually, it is experienced as an ordinary natural process, but also as a burden.

"You feel that you are on your own and have to do it all. The idea that it will never change, can be an enormous burden." [couple 3: female partner]

All partners mentioned how the disease effected their employment. Most partners had been able to adapt their working hours to their home circumstances, combining home and work tasks to support their partner.

#### If I did not do it, nothing happened

Partners experienced that they had to do it all: the household, the administration as well as initiating social events and keep in touch with friends and family. They noticed the difficulties their partners with MD1 had in initiating action. The inactivity and deferral of duties often was a frustrating experience for partners.

"What can be postponed was postponed. It completely irritated me. Then I thought come on, do something!" [couple 3: female partner]

#### I am in charge; I give directions

Partners found themselves completely in charge of what needs to be done, whereas in the past they had shared responsibilities. Several partners used reminders. One partner showed a list of duties for her partner which she wrote down every day, whereas another partner mentioned that she made telephone calls from work to prompt for actions. Partners noted the willingness of the person with MD1 to act, but the need for an extra stimulus to actually get going.

"I am often the one who plans everything. He usually agrees. Sometimes he is too tired for things. Then I have to say well come on. I write down the things he can do. I say you're at home all day, so you can also do something, stay busy, we must do it together." [couple 2: female partner]

Some partners deliberately gave directions based on their knowledge and understanding of the cognitive consequences of MD1. However, not all partners were aware of these. One partner felt that her partner's brain did not work well, as if there was some kind of dementia. She felt that she constantly had to say something, otherwise nothing would be done.

### Theme 3: Finding a mode together

Couples experienced living with MD1 as a process with dilemmas and frustrations, but compared it to the give and take required in every marriage. They felt ambivalence whether to do tasks themselves or encourage their partner to do it. Giving each other freedom and respecting differences in pace and interests were seen as important ways of managing together.

"I think it is important that you do it together. The one with the disease should not give up too easily, but should try to do what is possible. You have to find a good mode together." [couple 4: male partner]

MD1 leads to changes in roles and the marital relationship. Several partners indicated that they can imagine that marriages fail. A partner told how she had been jealous on other couples who happily did things together, while her husband always fell asleep. When she was 40, she felt being married to a man of 80. Couples also had to deal with changes in sexuality and intimacy in the relationship, which both experienced as difficult.

"You become a carer." [couple 1: female partner]

"We pretty much live like brother and sister." [couple 1: male with MD1]

#### Doing or leaving; renegotiation of tasks

Participants with MD1 said they want to do things themselves for as long as possible. They experienced that their partner sometimes gets impatient when performance of tasks takes much time. On the other hand they welcomed partner assistance when efforts became disproportionate for them or associated with safety risks. One participant mentioned this when she asked the interviewer to pour the tea from the teapot, as she experienced much difficulties doing this. Partners faced dilemmas when to encourage their partner to do activities themselves and when to take over. Couples indicated the importance for the person with MD1 remaining active even though doing so, demanded more time, effort or was clumsy.

"If it is difficult to rise from your chair because you have pain in your legs and your arms and you want to put on the potatoes. Then it is much easier to ask the other to do it..... For things I know that she cannot do it, I have no problems to take over. I get annoyed when I think if you try a little harder, you can do it yourself." [couple 4: male partner]

The balance between encouraging and taking over seemed to be variable and context-specific. A partner mentioned how she enjoyed watching her husband playing with their granddaughter. She knew this cost him energy, so she took over other activities for him. Previous roles and personal interests influenced this balance. In some couples there was consciously renegotiating of tasks, but there were also couples whereby nearly all tasks were gradually taken over by the partner.

#### Individual and shared activities; give each other freedom

Couples experienced an increasing difference between each other's capacities, pace and interests. They thought about what they could do together and what they could not. If they choose 'together', they adjusted the pace and enjoyed being engaged in shared activities.

"If we go shopping together, we do this leisurely at a snail's pace. Then it is not that I run to the supermarket after work, toss something in the car and fly home. No, we take the time and enjoy shopping together. We have chosen to do it this way." [couple 3: female partner]

All couples commented that going out was important for them. However, spontaneous outings or holidays had led to disappointment due to inaccessible public buildings. This meant that outings increasingly needed planning and preparation, usually done by the partners. Limited stamina, fatigue and sleepiness of persons with MD1 also influenced going out together because of the rest needed during the day.

"If we go away together, we are often confronted with a lot of problems. If you go to a museum, you might have to take six flights of stairs before reaching the entrance, then the game is over for us.... It means that you cannot go anywhere spontaneously." [couple 4: male partner]

Couples found solutions to engage in shared activities, like cycling with electric bikes in the surroundings, visiting accessible restaurants or taking a wheelchair to go out. Several spoke about enjoying just sitting together in their garden. They showed where they would sit together when the weather was nice. Couples emphasised the importance of giving each other freedom. Persons with MD1 were usually quite happy for their partner to do things for themselves. Partners learned that doing things for themselves is a way to re-energise and was experienced as taking good care of one self.

"I give my wife a lot of freedom. If she wants to go away with friends, that's fine with me. You go and have fun, I don't feel the need to go." [couple 3: male with MD1]

"Freedom is important, because at times it is quite heavy. A source of energy, whether it is sports or something else, gives you new energy, as long as you have something for yourself." [couple 2: female partner]

### Theme 4: Lack of understanding

Persons with MD1 reported feeling misunderstood and judged as silly or sulky, due to their facial weakness. Couples described a lack of understanding within their family, among relatives and friends and in the health care system and society at large. They speculated that was this was due to invisible problems like fatigue, but felt hesitant to ask for understanding, tending to withdraw from situations and hide their problems.

"Previously, I was the centre of attention. That is no longer the case and I have to get used to that. Now I am being looked at as if I am 'not quite in line'. I notice this and it annoys me." [couple 3: male with MD1]

"You constantly have to defend yourself and explain and I do not always feel like it. There are people who don't understand me and say 'yes' when it should be 'no'. That annoys me. I rather have them ask me to repeat myself than an answer that makes no sense ... then I withdraw..." [couple 5: female with MD1]

#### Even the family does not understand

Couples felt misunderstood by their own family. They experienced that relatives with the disease sometimes denied having MD1 and did not want to have anything to do with it. Partners even noted that the parent with MD1 showed little understanding for their own child(ren) with an even more severe form of the disease.

"When it was discovered by her mother, we told everyone to do a DNA test, then you know where you stand. If you see them walking, you can see it. Her own brothers do not know, because they did not do a test." [couple 5: male partner]

From relatives and friends they experienced lack of understanding of what it means to live with MD1. This led to hurtful reactions from others when for instance an appointment had to be cancelled due to ill-health or when they experienced that family members did not make efforts to understand their relative with less intelligible speech.

"When he takes the phone, they always ask for me, because they cannot understand him. And these are his own brothers. Or when they sit next to him, they don't have the patience to let him tell what he wants to say, that sort of things. He talks slowly and it gets worse." [couple 1: female partner]

#### We manage ourselves

Couples experienced that people are busy with their own lives and forget about them. They preferred to manage themselves and found it difficult to ask for support. Becoming dependent on others or receiving the 'wrong' assistance was a reason for giving up activities and withdrawing.

"We play 'jeu-de-boule' nearby. I cannot pick up a ball, but I have magnets to do that. [he showed how he managed with the magnets]. Then there are people that pick up the ball for me, all with the best intentions, but I hate that so much" [couple 3: male with MD1].

However, when support was given in the 'right' manner, it was highly appreciated. When friends showed understanding and consideration for their restrictions, this was treasured. One participant with MD1 mentioned that their group of friends had asked her to explain about her illness. When she had done so, she experienced better understanding.

"Last weekend we went to dinner with some good friends. We would have had dinner in a restaurant where the toilet was downstairs, but they then choose a different restaurant. They had thought about that which was fantastic." [couple 4: male partner]

#### Health care: many islands focused on part of the disease

Participants reported that they often visit many specialists. They felt little attention was paid to the consequences of the disease for their daily life. A couple described the hospital as 'many islands', each looking at one part of the disease. Couples said they visited the hospital together, because that way you hear more. One partner said that she did not mind doing personal care tasks but that she needed proper instructions and support. This couple expressed concerns about coping with a catheter. They wondered whether cycling, attending the choir or swimming was still possible. These questions had not been addressed in the consulting room with healthcare professionals, so they had tried to manage and find solutions themselves.

"You expect that they tell you these things [how to cope with a catheter] in hospital. Now I need to go to the drugstore. They have something for men who are incontinent. I have thought of it yesterday. You have to do some doctoring yourself. If they had only said how to do it, but they had not." [couple 1: female partner]

Some couples experienced understanding, recognition and support by fellow patients during meetings from the muscular disease association or group medical appointments, a 2 hour medical consultation together with 6 or 7 other patients with the same disease and their partners.

## Discussion

This is the first study exploring how couples live and cope with MD1. It offers new insights into the self-management strategies used by couples from the perspective of persons with MD1, their partner and as a couple. Persons with MD1 experienced physical, cognitive and social barriers in daily life. Their strategies included postponing, avoiding or giving up activities, but also adjusting expectations and finding alternative ways to engage in less demanding activities. Partners reported an increasing burden and felt that if they did not do things, nothing happened. Strategies used by partners included taking over activities and giving instructions and prompts. As a couple they faced challenges in finding a mode together with renegotiation of tasks and individual and shared activities. Giving each other freedom, respecting each other's differences in pace and giving scope for their own interests were ways in which couples managed together. Couples experienced lack of understanding from family, friends, society and health care. This lack of understanding contributed to further withdrawal and avoidance of social situations. Couples said they preferred to help each other, rather than asking for help or understanding. Health care was experienced as many islands, with each speciality looking at one part of the disease. Little attention was paid to the consequences of this systemic, neuromuscular disease for their daily life and marital relationship.

### Strengths and weaknesses of the study

Although a small, purposive sample (three men and two women with MD1), the strength of the study was that three in depth interviews, lasting between 60-90 minutes, were held with each couple. This resulted in 15 interviews with rich information from three perspectives: persons with MD1, their partners and as a couple. The codes saturated the four themes and resulted in "theme saturation" as well as "theoretical saturation"[28]. There were no obvious deviant or negative cases, but categories were saturated with a variety of positive and negative experiences. The trustworthiness of the study credibility was promoted through triangulation [25,29].

There was homogeneity in age and the type of MD with the onset of symptoms in adulthood. All participants were Caucasian. There is no information on how MD1 affects the lives of persons from other than western cultures. Younger persons with MD1 might have different experiences, especially since this disease has the characteristic of anticipation, meaning that children of couples with MD1 may have a more severe type of MD revealing problems at an earlier age. The findings give an impression of the lives of middle aged couples with one partner having MD1. These middle aged couples may have found a way to live with MD1, which may not be the case in younger couples, who are in a different phase in their lives. Therefore these findings cannot easily be generalised beyond older couples.

Besides, it is to be expected that there is a considerable group of men with MD1 without a partner. Men with MD1 appear to have a decline in marriage eligibility whereas women continue to marry at a young age and in a proportion almost equal to that of the unaffected population [30]. In a study of personality patterns of 15 people with MD1, 11 lived alone after divorce or were unmarried [5]. Our research shows the support needs of individuals with MD1 and support strategies provided by their partners. This support need probably also applies for single persons with MD1. This often causes an additional burden for the healthy parent who also takes on the responsibility of taking care of and encouraging their child(ren) with MD1.

This study also included participants with communication difficulties due to dysarthria in contrast to a previous qualitative study [8]. In some participants intelligibility was limited. The researcher repeated the participants' answers in order to make sure she understood; this repetition also facilitated the transcription.

Although MD1 is the most prevalent adult type of neuromuscular disease, the incidence is approximately 1 in 20.000; these findings may be relevant for couples with other complex chronic illnesses facing physical, cognitive and social impairments which limit everyday activities and social participation.

### Meaning of the study: possible explanations and comparison with other studies

Previous studies aiming to get insight in the consequences of MD1 used quantitative approaches [9,31-33], interviewed a heterogeneous group of persons with muscular dystrophies or their partners separately [8,12,34] or focused on living with the hereditary aspect of a muscular disease [13]. This study is the first to combine clients' and partners' perspectives on their daily life experiences and how they cope as couple with MD1.

Lack of understanding of family, friends, society and health care was a major theme which was described in other studies in muscular dystrophy [8,11,12,35,36]. The following factors may be possible explanations: lack of communication, wrong expectations, ignorance and avoidance. Communication was not only an issue because of dysarthria, decreased intelligibility and limited facial expression in persons with MD1. More crucial is the lack of communication about the illness experience of persons living with MD1. How can friends, family and health care professionals understand if they do not communicate about the impact of the disease on daily life? Although patient-centred care is being promoted [37], participants in current study still experienced illness-centred care with many health care professionals focusing on part of the disease and its management and not on the consequences for their daily life. A recent review on the management of MD1 confirmed the concentration on impaired functions and structures in medical subsystems [38].

Other factors contributing to lack of understanding include wrong expectations and ignorance of other people regarding the potential or limitations of persons with MD1. As MD1 is classified as a neuromuscular disease, muscle weakness and fatigue are expected and treatment is aimed at improving body functions like aerobic capacity, breathing or hand function [14,17,18,39,40]. However, the additional cognitive and social barriers may explain why adherence to exercise programs is limited [14]. Breaking down disabling social practices against people with MD1 might be as important, if not more so, than seeking treatment for physical impairments [41]. This is in accordance with Boström and Ahlström who state that: many issues in the management of long-term chronic illness can better be interpreted from a social perspective rather than a biomedical perspective [34].

Professionals and others primarily focus on the needs of the person with MD1 and overlook the support needs of partners or other family members [12]. Health professionals usually perceive partners as caregivers rather than care recipients. Also partners do not express their need for support, although they might experience a large caregiver burden, feeling no scope for own interests outside the home which can be a source of despair [12]. This study showed how frustrating and annoying the lack of initiative and avoidant behaviour can be for partners. This is in agreement with findings from Timman et al. who found that for partners, worse general well-being and more anxiety was associated with a lack of initiative of the their partner with MD1 and less marital satisfaction[42]. Partners experience difficulties in interpreting whether the need for support is related to MD1 or to lack of will or laziness [12]. Not all partners were aware that lack of initiative and avoidant behaviour are part of MD1. Current study showed that insight and awareness that these problems are related to MD1 helped to more consciously apply compensation strategies like prompts or reminders.

Current study also showed the resilience of couples to manage together. Supporting each other and giving each other freedom were described as vital couple management strategies. This finding replicates the management strategies used by couples facing other multiple chronic illnesses [43]. Two life philosophies were found: 1) staying positive; and 2) being married means supporting each other whatever happens and coping with what comes along. The combination of individual and shared coping strategies was considered indicative of a healthy balance [43]. A continuum was described between positive support like a helpful reminder and problematic support like nagging or pushing a partner [43]. A balance was promoted in which the person with the disease manages themselves and the partner provides support to this managing. How assistance is given and received as well as marital interactions that accompany this support, appeared to impact both marital quality and health [43]. These findings support a couple-centred approach in couples with complex chronic diseases like MD1.

A couple management approach has shown to be effective for patients with dementia and their caregivers [44,45]. The problems experienced by these couples in relation to coping with loss of abilities, initiative and participation in social activities, decreased quality of life and pressure on family relations and friendships [44] are strikingly similar to the problems experienced in MD1. The consequences of MD1 have been described as comparable to those of the aging population [15]. This is in line with insights that MD1 is considered a progeroid syndrome with accelerated emergence of features of senescence including symptoms of dementia [46]. There is evidence that a community approach aimed at increasing abilities of older people with dementia to engage in meaningful daily occupations and interventions to increase support giving skills of partners increases participation of the person with dementia and reduces caregiver burden [44,45]. Further studies evaluating couple management for couples with other progressive, complex diseases are warranted.

### Implications for clinicians and policymakers

Although there is mounting evidence for effective system changes that improve chronic care, such as the Chronic Care Model [47-49], health care often remains with basic structures and practices designed for acute diseases [50]. The traditional medical care model mainly focuses on treatment of impairments [6], ignoring the illness experience and its impact on the system, including partners. Our recommendations for clinicians therefore include:

1. A shift from the focus on physical functions to a person-, or even a couple-centred approach aiming to understand not only the disease, but also the illness experience of persons with a complex chronic illness and their partners. This shift involves:

- A more narrative approach besides the traditional biomedical analytical approach; and

- Referral of patients and their partners to appropriate interdisciplinary medical, rehabilitation and community services to address barriers from medical as well as psychological and social perspectives.

2. A shift from self-management to couple management in complex chronic conditions, in which self-management skills of both partners are supported in order to:

- Maximize participation for persons with a complex chronic disease such as MD1;

- Reduce caregiver burden by enhancing support giving skills; and

- Find a healthy balance together in shared and individual activities.

### Future research

Challenges for the future include further development and evaluation of the acceptability and effectiveness of a couple-centred approach for complex chronic illnesses. A couple-centred approach includes the three aspects of self-management: medical management, role management and emotional management [51] and should also address disabling social practices. As this can be considered a complex intervention including several components [52,53], it is recommended to use a stepwise approach as described in the framework for design and evaluation of complex interventions [52].

## Conclusions

Persons with MD1 experience physical, cognitive and psychosocial barriers in the performance of activities and participation in life roles, leading to postponing, avoiding, adapting or giving up activities. Partners experienced an increasing burden and felt that they had to do it all, including prompting their partner to act. For couples this led to changes in tasks, responsibilities and relationship. Couples described a lack of understanding within their family, among relatives and friends, in the health care system and in society at large. Health care was perceived as fragmentary, with specialists focusing on specific aspects of the disease. This study showed how important it is to listen to couples, to elicit the impact of MD1 for daily life. An integrated medical and social approach for persons with MD1 is recommended. Couple management, supporting the self-management skills of both partners is proposed as a way of reducing the mismatch between health services and health needs.

## Abbreviations

MD1: myotonic dystrophy type 1.

## Competing interests

The authors declare that they have no competing interests.

## Authors' contributions

EHCC designed the research, did the interviews and transcribed, analysed and interpreted these and drafted and revised the paper. AK and TS designed the research, analysed and interpreted the interviews and drafted and revised the paper. AJP, HTH and RABO interpreted the interviews and drafted and revised the paper. GJvdW and BGMvE designed the research, analysed and interpreted the interviews and drafted and revised the paper. GJvdW and BGMvE contributed equally. All authors approved the final version to be published.

## Pre-publication history

The pre-publication history for this paper can be accessed here:

http://www.biomedcentral.com/1471-2377/11/86/prepub
